# Anti-Neuroblastoma Properties of a Recombinant Sunflower Lectin

**DOI:** 10.3390/ijms18010092

**Published:** 2017-01-10

**Authors:** Marcela Pinedo, Melanie Genoula, María Ximena Silveyra, André De Oliveira Carvalho, Mariana Regente, Marianela Del Río, Júlia Ribeiro Soares, Valdirene Moreira Gomes, Laura De La Canal

**Affiliations:** 1 Instituto de Investigaciones Biológicas (IIB), Consejo Nacional de Investigaciones Científicas y Técnicas (CONICET), Universidad Nacional de Mar del Plata, Funes 3250, 7600 Mar del Plata, Argentina; mgenoula@gmail.com (M.G.); mxsilveyra@gmail.com (M.X.S.); mregente@mdp.edu.ar (M.R.); maris03_5@hotmail.com (M.D.R.); ldelacan@mdp.edu.ar (L.D.L.C.); 2 Laboratório de Fisiologia e Bioquímica de Microrganismos, Centro de Biociências e Biotecnologia, Universidade Estadual do Norte Fluminense, Campos dos Goytacazes 28013-602, Brazil; andre@uenf.br (A.D.O.C.); julliah@hotmail.com (J.R.S.); valmg@uenf.br (V.M.G.)

**Keywords:** agglutinin, *Helianthus annuus*, Helja, jacalin, neuroblastoma, recombinant lectin, tumor toxicity

## Abstract

According to their sugar recognition specificity, plant lectins are proposed as bioactive proteins with potential in cancer treatment and diagnosis. Helja is a mannose-specific jacalin-like lectin from sunflower which was shown to inhibit the growth of certain fungi. Here, we report its recombinant expression in a prokaryotic system and its activity in neurobalstoma cells. Helja coding sequence was fused to the pET-32 EK/LIC, the enterokinase/Ligation-independent cloning vector and a 35 kDa protein was obtained in *Escherichia coli* representing Helja coupled to thioredoxin (Trx). The identity of this protein was verified using anti-Helja antibodies. This chimera, named Trx-rHelja, was enriched in the soluble bacterial extracts and was purified using Ni^+2^-Sepharose and d-mannose-agarose chromatography. Trx-rHelja and the enterokinase-released recombinant Helja (rHelja) both displayed toxicity on human SH-SY5Y neuroblastomas. rHelja decreased the viability of these tumor cells by 75% according to the tetrazolium reduction assay, and microscopic analyses revealed that the cell morphology was disturbed. Thus, the stellate cells of the monolayer became spheroids and were isolated. Our results indicate that rHelja is a promising tool for the development of diagnostic or therapeutic methods for neuroblastoma cells, the most common solid tumors in childhood.

## 1. Introduction

Lectins are proteins that specifically bind different sugar motifs due to their carbohydrate recognition domains [1]. This ability allows them to interact with glycoproteins and glycolipids, fulfilling diverse functions related to cell recognition [2,3]. As they can recognize the carbohydrate code on different cell surfaces, they might distinguish tissue types and the occurrence of variations in the exposed glycocode [4]. It must be taken into account that the malignant transformation of cells toward the invasive behavior leading to metastasis has been associated with complex alterations in the glycosylation process, which, in turn, can produce changes in cell–cell and cell-extracellular matrix (EM) interactions and favors the progression of the tumors [5,6]. Thus, the binding properties of the lectins allow their use in the diagnosis and treatment of cancer disease [4,7,8], and it is not surprising that a range of biomedical applications has been described for different lectins [1,8]. In fact, a significant number of papers and patents use lectins as diagnostic tools [9].

Plant lectins have attracted special attention because of their abundance in seeds and ease of preparation. They have been shown to bind specifically to cancer cell membranes or receptors, causing inhibition of tumor growth [4,10,11]. In addition, their differential recognition of cells may also be viewed as an essential part of the mechanism required for the targeted delivery of drugs [12]. Some plant lectins also reap the benefit of antifungal and antiviral activities [13,14,15]. However, it was claimed that their range of applications needs to be improved with the availability of novel lectins with differential specificity and biological targets [8]. Nevertheless, it must be taken into account that the thorough analysis of the biological properties is necessary to exclude harmful consequences that may arise *in vivo* by the administration of lectin-containing extracts [16].

We have previously characterized Helja, a lectin isolated from sunflower seedlings which exhibits mannose specificity and belongs to the Jacalin family [17]. This lectin was shown to inhibit the growth of human pathogenic fungi [14]. However, the exploration of other putative activities was limited by the low abundance of Helja in its natural source. Hence, we decided to conduct its recombinant expression taking advantage of the absence of post-translational modifications in native Helja [18] that allowed the use of a bacterial platform.

The aim of this work was to produce an active recombinant form of the jacalin-like lectin Helja and test its effect on SH-SY5Y neuroblastoma cells. Neuroblastomas account for 15% of cancer related deaths in children and represent the most common extracranial solid tumor during childhood [19]. The surface of these cells exhibit glycoproteins decorated with mannose residues [20,21,22] and might hence be a target of Helja that would allow the use of the lectin in diagnosis or therapy of neuroblastoma.

## 2. Results and Discussion

### 2.1. Expression of the Recombinant Jacalin-Like Lectin Helja

**Figure 1:**
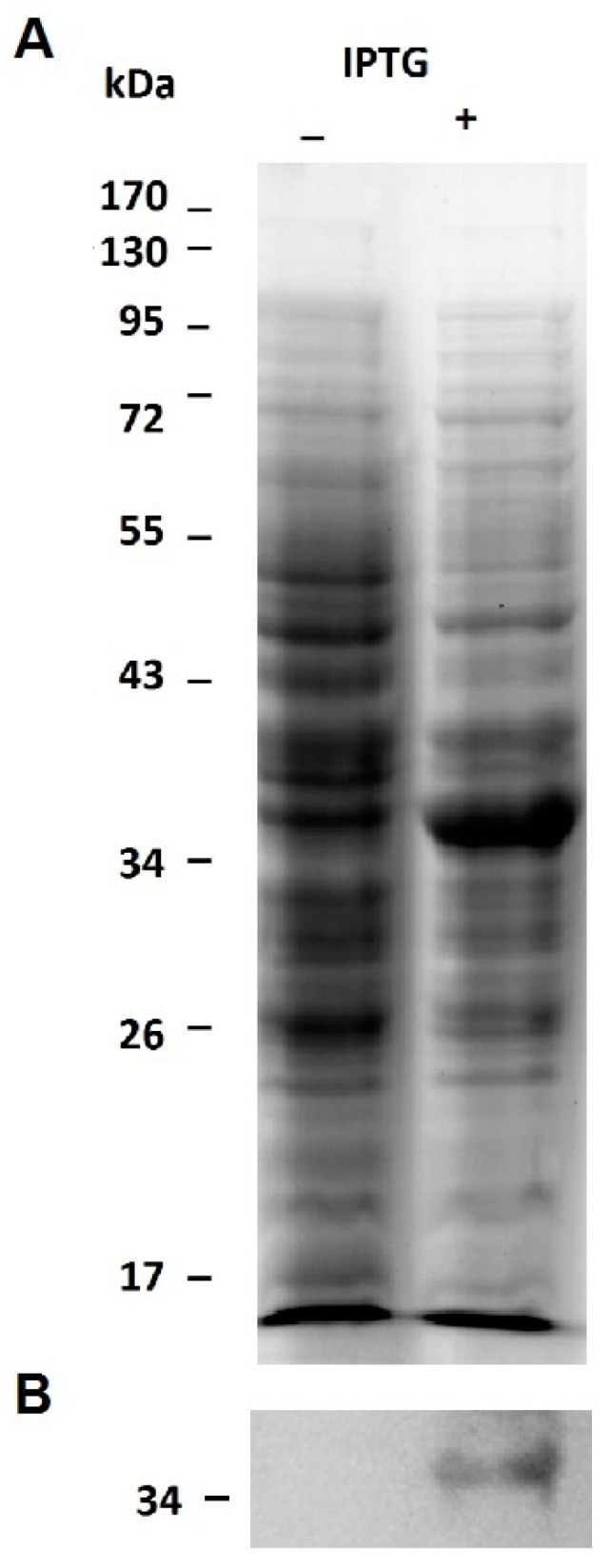
Induction of recombinant Trx-rHelja expression. The protein profile of extracts was obtained after lysis of cells from un-induced (−) or isopropyl β-d-1-thiogalactopyranoside (IPTG) induced (+) *E. coli* cultures. The extracts obtained from 0.1 mL of cell cultures with an OD at 600 nm of 1.5 were loaded on a 12% denaturing polyacrylamide gel electrophoresis, SDS-PAGE, and subsequently stained with Coomassie Brillant Blue (**A**). Immunodetection of Trx-rHelja in extracts of 0.01 mL of un-induced (−) or IPTG induced (+) cell cultures. Gel performed as described above was transferred to nitrocellulose, blocked and incubated sequentially with 1:3000 anti-Helja polyclonal antibodies and alkaline phosphatase-conjugated anti-rabbit IgG 1:10,000 (**B**). Figures are representative of three (*n* = 3) independent experiments. Molecular mass markers are indicated on the left.

A problem frequently encountered during the production of recombinant proteins in *E. coli* is the occurrence of insoluble and/or non-functional forms of those proteins. To overcome this limitation, we decided to express Helja as a fusion protein with thioredoxin since it increases the solubility of heterologous proteins synthesized in *E. coli* cytoplasm [23]. Helja amino acid sequence was previously deduced by cloning and sequencing the complete coding sequence (CDS) [24]. Therefore, in this work, Helja CDS was cloned in the expression enterokinase/Ligation-independent cloning vector pET-32Ek/LIC to obtain a chimeric thioredoxin-Helja protein (Trx-rHelja) in the *E. coli* Rosetta-gami2 (DE3) pLysS expression strain. Subsequent vector sequencing confirmed that Helja CDS was in frame with the ORF of the vector containing the His-tag and the enterokinase cleavage site upstream the methionine 1 of Helja (Figure S1). In order to obtain the recombinant protein, isopropyl β-D-1-thiogalactopyranoside (IPTG) induced cells were lysed by sonication, and the protein extracts were compared with those of un-induced cultures using 12% denaturing polyacrylamide gel electrophoresis SDS-PAGE. Figure 1A shows the occurrence of a band of approximately 35 kDa enriched in the 0.5 mM IPTG treated cultures. This MW is in agreement with the fusion of Helja to thioredoxin (Trx) and the His tags plus the entorokinase recognition sequences present in the vector. To validate the identity of the induced protein, an immunoblot was done using antibodies raised against Helja purified from sunflower seedlings [17]. The band of 35 kDa, immunodetected in the membrane showed in Figure 1B, confirmed the occurrence of the jacalin-like lectin, now on named Trx-rHelja. This result supports the fact that the recombinant protein was induced and translated in the correct frame in the prokaryotic expression system.

The expression conditions were further optimized to increase Trx-rHelja production and solubility. The induction with 0.5 mM IPTG with a growing temperature of 30 °C was found to be the best mode to produce the soluble recombinant protein. Under these conditions, the 35 kDa induced protein was enriched in the 10,000 g soluble fraction (SN) when it was compared to the pellet (PP) obtained from *E. coli* extracts (Figure S2). Therefore, it was concluded that the overall process of Trx-rHelja production was successful.

### 2.2. Purification of the Recombinant Helja

**Figure 2:**
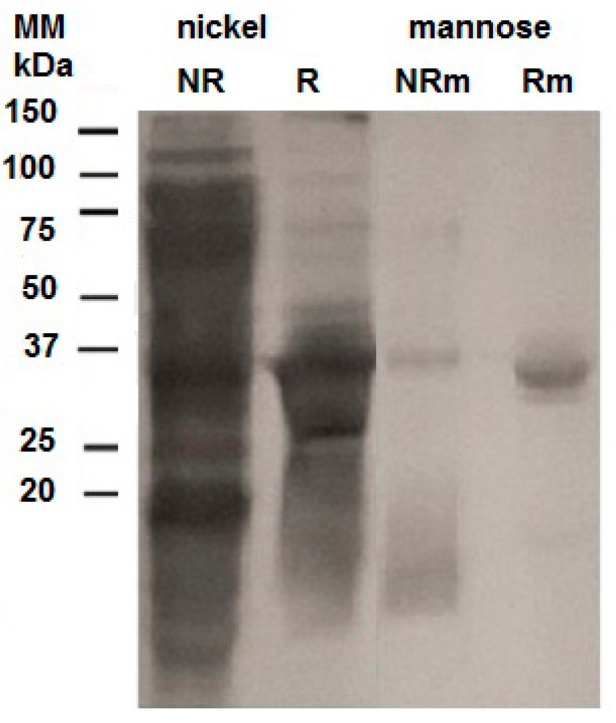
Recombinant Trx-rHelja purification by affinity chromatography. The protein profile of samples collected during Trx-rHelja purification scheme was analyzed using sequentially affinity for Ni^2+^-Sepharose and mannose-agarose matrixes. The soluble fraction (SN) of extracts from 100 mL IPTG induced *E. coli* cultures were loaded on the metal affinity matrix and the non-retained (NR) or the retained (R) fractions were recovered. Then, R was loaded on a mannose-agarose column and the proteins, the non-retained (NRm), and retained peaks (Rm) were recovered. Aliquots of R, NR, Rm and NRm fractions were analyzed in 12% SDS-PAGE and subsequently stained with Coomassie Brillant Blue. Figure is representative of two (*n* = 2) independent experiments. Molecular mass markers are indicated on the left.

The presence of six histidine residues linking the thioredoxin and the lectin domains allowed the isolation of Trx-rHelja using a metal affinity chromatography. Thus, the SN fraction of induced cultures was loaded on a Ni^2+^-Sepharose matrix and the retained (R) proteins were eluted with 150 mM imidazole. Both the non-retained (NR) and the R fractions recovered from the matrix were analyzed by SDS-PAGE. The R fraction was enriched in a protein band of the expected molecular mass for Trx-rHelja (Figure 2). Since other proteins were also evident in this fraction, an affinity chromatography with d-mannose attached to agarose resin was used to further purify Trx-rHelja. The SDS-PAGE analysis clearly demonstrated that the recombinant protein was bounded to the matrix and eluted with mannose (Figure 2). Therefore, this procedure not only was useful for the purification of the recombinant protein but also showed that Trx-rHelja appears to have a sugar-binding capability similar to the native Helja even if it is still fused to the Trx and His tags. It also indicates that the lectin was correctly folded in the cytoplasm of *E. coli*, as the mannose binding activity depends on the conformation properties.

Other lectins successfully expressed in bacteria include the lectins from the blue-green algae *Microcystis viridis* called MVL [25], the jacalin lectin CRLL from leaves of *Cycas revoluta* [26] and frutalin from *Artocarpus incised* [27]. However, when *E. coli* was used to produce the recombinat jacalin from *Artocarpus intergrifolia*, the obtained chimera had reduced carbohydrate binding affinity compared to the native form. The authors justified this later result on the necessity of the posttranslational processing required for the protein [28].

**Figure 3:**
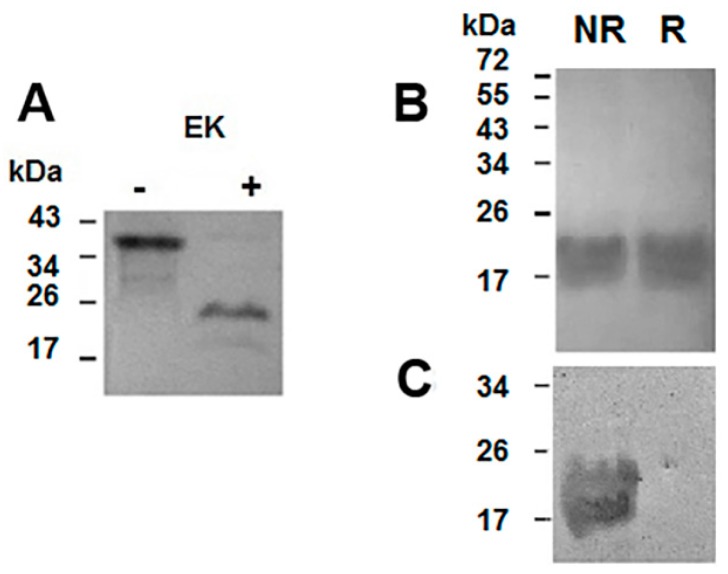
Trx-rHelja digestion with enterokinase and rHelja purification. Aliquots of the mix containing enterokinase after (+) and before (−) of the incubation were loaded on 12% SDS-PAGE and subsequently transferred to nitrocellulose, blocked and incubated sequentially with 1:3000 anti Helja polyclonal antibodies and 1:10,000 alkaline phosphatase-conjugated anti-rabbit IgG (**A**). Aliquots of the proteins from the non-retained (NR) or the retained (R) peaks obtained after loading the products of the enterokinase digestion on the Ni^2+^-Sepharose matrix were analysed in a 12% SDS-PAGE and stained with Coomassie Brillant Blue (**B**) or transferred to nitrocellulose, blocked and incubated sequentially with 1:3000 anti Helja polyclonal antibodies and 1:10,000 alkaline phosphatase-conjugated anti-rabbit IgG (**C**). Figures are representative of three (*n* = 3) independent experiments. Molecular mass markers are indicated on the left.

The release of Helja from the fused tags was performed through the incubation with enterokinase and the products of this reaction were evaluated in gels. Thus, the immunoblot assay of Figure 3A shows that the enzyme significantly reduced the 35 kDa band, representing the recombinant protein, and gave rise to a protein of around 20 kDa, the expected size for Helja. The immunoreactivity of the 20 kDa band supports the fact that it was the desired recombinant jacalin like lectin, now on named rHelja. rHelja was isolated by loading the reaction mix on the Ni^2+^-affinity column (Figure 3B,C) and, in accordance with the absence of the histidine tag, it was recovered in the washing buffer. Finally, this fraction containing rHelja was exhaustively dialyzed against PBS and subsequently concentrated to test its bioactivity.

### 2.3. Helja Activity on Human Neuroblastomas

**Figure 4:**
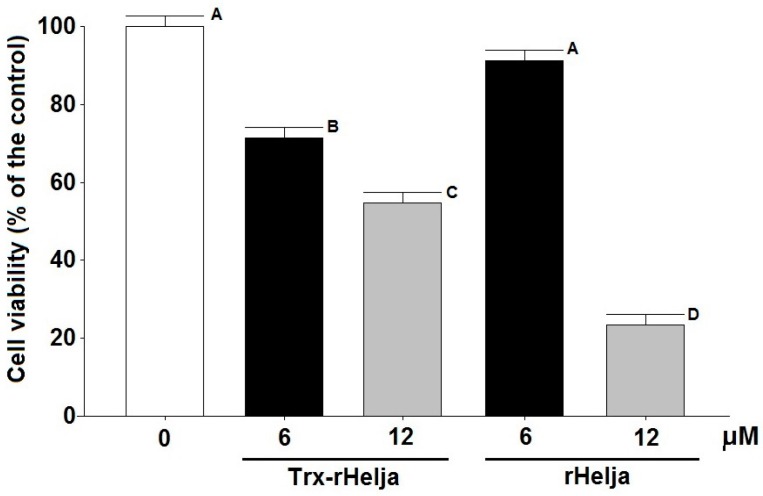
Cell viability of human SH-SY5Y neuroblastoma cells treated with Trx-rHelja or rHelja. The cells at density of 5 × 10^4^ cell/well were grown for 24 h before the incubation with 6 and 12 µM Trx-rHelja or rHelja. After an additional 24 h, MTT reagent was added to the wells. Dimethyl sulfoxide was used to dissolve the formazan complex and the absorbance was measured at 570 nm. The cellular viability was expressed as a percentage of the value obtained in the absence of treatment. Each experiment was tested in triplicates and they were repeated at least three times. Standard deviation, one-way ANOVA and Tukey test analysis (*p* ≤ 0.05) were performed using InfoStat software (2014, Agricultural College of the National University of Córdoba, Córdoba, Argentine). Different capital letters indicate significant differences.

Trx-rHelja and the enterokinase-released rHelja were assessed for their effect on human neuroblastoma SH-SY5Y tumor cells using confluent cultures incubated for 24 h with 6 and 12 μM of both lectins. After incubation, the cells were analyzed for their viability through an 3-(4,5-dimethylthiazol-2-yl)-2,5-diphenyltetrazolium bromide (MTT) standard assay. Viable cells reduce MTT to a purple formazan that can be quantified by a spectrophotometric approach. The diminution of the formazan concentration observed in the lectin treated cells relative to the controls clearly supports the cytotoxic action of the lectin on the SH-SY5Y cell line (Figure 4). The incubations with 6 and 12 μM of the recombinant Trx-rHelja decreased cell viability to approximately 75% and 55%, respectively, and rHelja also showed a dose-dependent diminution of cell viability. Only 25% of cells remained alive upon incubation with the highest rHelja concentration (12 μM or 200 μg/mL).

The toxicity observed for rHelja is similar to the native and recombinant forms of frutalin, which is a α-d-galactose specific jacalin able to affect HeLa cells with an IC_50_ of 100 μg/mL [29]. Similarly, the antitumoral activity for a lectin isolated from Del Monte banana, inhibited a 40%–60% the proliferation of leukemia cancer cells L1210 at concentrations among 80–500 μg/mL [30].

Plant lectins with antitumoral activity have been recently reviewed [4,7] and a few of them have reached clinical trials. Bioactive molecules with potential as anticancer molecules include the *Polygonatum odoratum* lectin (POL) and concanavalin A (Con A). POL is a d-mannose specific protein of the B-lectin family with 50% inhibitory rate on fibrosarcoma cells at 23–25 μg/mL [8]. Meanwhile, Con A produces 60%–90% inhibition of human melanoma A375 cells when tested at 80 μg/mL [10]. The demonstration of Helja cytotoxicity on neuroblastoma cells shown in this report acquires a particular interest since, according to recent reviews and bibliographic searches, plant lectins reported to be active against neuroblastoma are scarce [4,7,8,31]. Here, we show that Trx-rHelja as well as rHelja induce the loss of the viability of neuroblastoma cells and support the idea that different versions of Helja are functional, even with the added tags, which might facilitate future applications.

**Figure 5:**
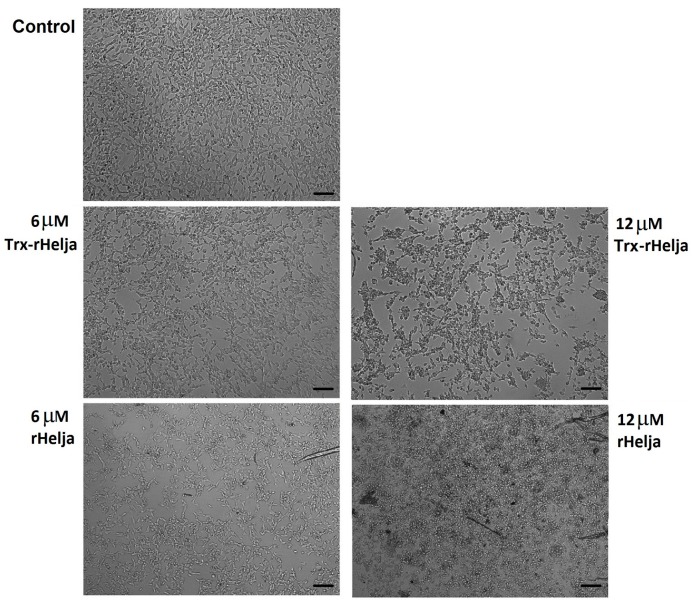
Cell morphology changes in human neuroblastoma SH-SY5Y cells treated with Trx-rHelja or rHelja. The cells at density of 5 × 10^4^ cell/well were grown for 24 h before the incubation with 6 and 12 µM Trx-rHelja or rHelja for additional 24 h. Cells were examined using a phase contrast microscopy (10× lens). Scale bar = 10 μm.

The observation of neuroblatomas by optical microscopy provided more insight concerning rHelja action. Figure 5 shows significant morphological changes of the cells upon treatment. At the highest concentration tested (12 µM), neuroblastoma cells exhibited a retraction of their neurite extensions. The cells acquired a typical apoptotic appearance. They changed from a monolayer of stellate adherent cells to isolated spheroid shaped cells, of smaller size and without the neuritis connections [32]. Taking into account the important role that carbohydrates bound to proteins and lipids of the cell surface in cell–cell interactions and adhesion with the EM play [33,34], it can be hypothesized that Helja could disrupt the EM through interaction with cell surface sugar residues, thereby affecting the network of cells and their morphology. Indeed, CD24 and different isoforms of NCAM (NCAM-140 and NCAM-180), which are glycoproteins decorated with mannose residues that occur on the surface of neuroblastoma cells and are reported to be involved in cell adhesion [20,21].

Plant lectins can induce cytotoxicity, apoptosis and necrosis in various tumor cells using different mechanisms [11,35,36,37]. Some trigger apoptosis through the induction of specific caspases [11,38], while others, such as the mannose-binding lectins from *Canavalia ensiformis* (ConA) and *Canavalia brasiliensis* (ConBr), are able to make it through depolarization and permeabilization of mitochondrial membranes and accumulation of reactive oxygen species (ROS) [39]. Even though the basis of the cytotoxic activity of rHelja is not addressed in this work, it is tempting to speculate that oxidative stress could mediate neuroblastoma cell death since we have previously demonstrated that Helja induces ROS accumulation in other cells [14].

It has been recognized that it is imperative to conduct lectin research to identify which proteins have the greatest prospects against malignant cell lines threatening human health [8]. The results obtained in this study allowed us to exceed the limits imposed by the reduced availability of Helja in sunflowers. Expression in a prokaryotic system generated quickly and at low cost the soluble and active Trx-rHelja protein that, in turn, makes possible the production of rHelja. Both the fusion protein and rHelja are bioactive lectins cytotoxic against neuroblastoma. Therefore, Helja emerges as an alternative molecule with potential to be used as anticancer drug or in diagnosis, where the biopsy of tissues is an absolute requirement.

## 3. Material and Methods

### 3.1. Biological Material

Chemically competent cells of *Escherichia coli* bacterial strain NovaBlue with relevant genotype: *endA1 hsdR17* (r_K12_^−^ m_K12_^+^) *supE44 thi-1 recA1 gyrA96 relA1 lac*F′(*proA^+^B^+^ lacI^q^ Z*Δ*M15*::Tn*10*) (Tet^R^) were used for general cloning and cells of Rosetta-gami2 (DE_3_) pLysS with relevant genotype: Δ(*ara*–*leu*)*7697* Δ*lacX74* Δ*phoAPvuIIphoRaraD139ahpCgalEgalKrpsL* (DE_3_) F’[*lac*^+^*lacI*^q^*pro*] *gor522*::Tn*10trxB* pLysSRARE23 (Cam^R^, Str^R^, Tet^R^)^4^) for the expression of the sequence fused in the pET-32 EK/LIC, the enterokinase/Ligation-independent cloning vector (cloning kit, Novagen, Applied Biosystems, Foster City, CA, USA).

Human neuroblastoma cell line SH-SY5Y ATCC^®^ Catalog No. CRL-2266™ was kindly provided by Dr. Tomás Falzone (Instituto de Biología Celular y Neurociencia “Prof. E. de Robertis”, Buenos Aires).

### 3.2. Construction of the Recombinant Expression Vector

The set of specific primers for amplification of the Helja coding sequence intended to directionally clone the sequence in the pET-32 EK/LIC vector was designed according to the vector instruction manual (pET System Manual, Novagen). The complete coding sequence of Helja was previously obtained, GenBank KJ681498 [24]. The forward primer was 5′-GACGACGACAAG*ATGGCTAACAACTACGTTGAGG*-3′ and the reverse was 5′-GAGGAGAAGCCCGGT*CTAGGGACTAAGTACGA*-3′. Underlined letters correspond to the sequence that anneals with the pET-32 EK/LIC vector and the italic letters to the sequence that anneals with Helja cDNA. Primers were synthesized by Invitrogen (Carlsbad, CA, USA).

Fragment amplifications were performed by PCR containing 1× *Taq* buffer with (NH_4_)_2_SO_4_ (Fermentas), 1.5 mM MgCl_2_, 0.2 mM dNTP, 1 μM forward primer, 1 μM reverse primer, 0.2 μL cDNA obtained from sunflower cotyledons and 1.25 units Taq DNA polymerase I in a volume of 20 μL. The program used for amplification was 95 °C for 3 min, followed by 1 cycle of 95 °C for 45 s; 58 °C for 45 s and 72 °C for 1.5 min and 35 cycles of 95 °C for 45 s; 68 °C for 45 s and 72 °C for 1.5 min (Verit Thermal Cycle, Applied Biosystems, Foster City, CA, USA). The PCR product was purified using the Wizard SV gel (Promega, Madison, WI, USA) and PCR clean up system (A9281 Promega, Madison, WI, USA) and treated with T4 DNA polymerase to generate the compatible overhang ends to be annealed the pET-32 EK/LIC. The pET-32 EK/LIC vector containing Helja cDNA sequence was named pET-Helja and was used for bacterial transformation.

### 3.3. Transformation, Positive Colony Screening and Sequencing

NovaBlue competent cells, transformed with pET-Helja were grown in liquid Luria–Bertani (LB) medium. A colony screening was done by plasmid extraction (isolated using Highway ADN PuriPrep kit from INBIO, Buenos Aires, Argentina). It was followed by a restriction analysis with *Bgl* II and *Eco* RI as described in pET System Manual 11th Edition (Applied Biosystems, Foster City, CA, USA). Plasmids recovered from positive colonies were used to transform the expression strain Rosetta-gami2 (DE_3_) pLysS competent cells [40], which were grown in the presence or the antibiotics ampicillin (Sigma, St. Louis, MO, USA) and chloramphenicol (Calbiochem, Billerica, MA, USA), at 50 and 34 µg/mL, respectively. Colony PCRs identified positive colonies and three of them that were randomly selected were sequenced in both directions (Instituto de Biotecnología-Unidad de Genómica- INTA Castelar, Castelar, Argentina) using the forward and reverse universal T7 primers.

### 3.4. Expression of the Recombinant Lectin Helja in E. coli

Liquid cultures of positive colonies with an OD at 600 nm of 0.5 were induced with 0.5 mM of isopropyl-β-d-thiogalactopyranoside (IPTG, Sigma) at 30 °C until they reach an OD higher than 1.4. These conditions resulted upon optimization assays performed according to the manufacturer instruction manual testing different IPTG concentration (0.2–0.5 mM), and growth temperature (25–37 °C) to produce the higher relative amount of the recombinant protein in the soluble cell fraction. The cells, centrifuged at 10,000× *g* for 10 min at 4 °C, were re-suspended and lysed by sonication in buffer 50 mM HCl-Tris pH 8 and 100 mM NaCl, (200 µL buffer: 1 mL culture) with 15 bursts of 30 s (Microson XL, Misonix, Farmingdale, NY, USA). After centrifugation, the supernatant (SN) and the pellet (PP) were recovered for electrophoretic analysis.

### 3.5. Purification of the Recombinant Lectin in Ni^2+^-Sepharose and Mannose-Agarose Matrixes

Forty mL of SN, (correspondent to 2000 mL of cell culture) were loaded on 4 mL of the Ni^2+^ affinity matrix cOmplete His-Tag Purification Resin (Roche 05893682001, Pleasanton, CA, USA) previously equilibrated with buffer A (50 mM Tris-HCl pH 8 and 200 mM NaCl). They were incubated at 4 °C for 3 h with mild agitation before exhaustive washing of the non-retained proteins (NR) with buffer A. The retained fraction (R) was eluted with 15 mL of buffer A supplemented with 150 mM imidazole at a flow rate of 30 mL/h. The OD at 280 nm of the NR and R fractions was checked. The R fraction was loaded on 3 mL of d-mannose-agarose resin (Sigma M6400) equilibrated with buffer B (50 mM Tris-HCl pH 7.5, 100 mM NaCl). Non-bound proteins were washed before the elution of retained proteins with 12 mL of buffer B supplemented with 200 mM mannose according to Pinedo et al. [17]. When necessary, fractions were concentrated by acetone precipitation to be analyzed by SDS-PAGE.

### 3.6. Digestion of the Recombinant Protein

The recombinant protein named Trx-rHelja, has three parts: the thioredoxin tag at the N-terminal (Trx) followed by six consecutive histidines, a glutathione tag and the lectin (rHelja) at the C terminus. All tags can be eliminated by EK digestion. To perform EK digestion, 750 μg of the Trx-rHelja were incubated with 0.016 U of recombinant bovine EK (Sigma) for 16 h at 25 °C in buffer 50 mM Tris-HCl pH 8.0; 2 mM Ca_2_Cl and 1% *v*/*v* Tween 20 (volume 500 µL). The digestion products were evaluated in gels. The rHelja was purified on the Ni^2+^ affinity matrix as it was described above. Millipore Centricon^®^ Centrifugal Filter Units (cut-off 3 kDa) (Billerica, MA, USA) were used for protein concentration and buffer exchange. To limit the interaction of the protein with the filter membrane, glycerol in PBS was added till 5% final concentration.

### 3.7. Protein Analysis and Western Blotting

Gels that were 12% SDS-PAGE, performed according to Laemmli [41], were stained with Coomassie Brillant Blue R-250. PageRulerTM Prestained Protein Ladder (Fermentas, Life Sciences, Waltham, MA, USA) and Precision Plus Protein™ Kaleidoscope™ Prestained Protein Standards (Biorad #1610375, Hercules, CA, USA) were used as Molecular Mass markers.

The proteins were transferred to 0.45 μm nitrocellulose membranes, and incubated in blocking buffer (5% defatted milk in 0.1 M HCl-Tris pH 8.0) followed by the addition of anti Helja primary antibodies diluted 1/3000. After overnight incubation, the blot was revealed as described elsewhere [25] using anti-rabbit IgG conjugated with alkaline-phosphatase diluted 1/10,000 (Sigma, St. Louis, MO, USA).

Protein content was quantitated by the bicinchoninic acid method [42] adapted to microplate using bovine serum albumin (Sigma) as the standard.

### 3.8. Neuroblastoma Cell Culture

Human neuroblastoma cell line SH-SY5Y was maintained in complete Dulbecco’s Modified Eagle’s Medium (DMEM, Sigma) supplemented with 10% fetal bovine serum (FBS, Internegocios-Argentina, Mercedes, Buenos Aires, Argentina), 100 U/mL penicillin, 100 μg/mL streptomycin and 0.25 μg/mL amphotericin B (antibiotic-antimycotic solution from Sigma), at 37 °C in a humidified incubator with 5% CO_2_.

### 3.9. Cell Viability Assay

The viability of the neuroblastoma SH-SY5Y cells treated with Trx-rHelja and rHelja- was determined by the 3-(4,5-dimethylthiazol-2-yl)-2,5-diphenyltetrazolium bromide assay (MTT) according to Mossmann [43] with modifications. Briefly, cells were seeded in 96-well plates at density of 5 x 10^4^ cells/well and incubated for 24 h in DMEM supplemented with 10% (*v*/*v*) FBS. At that point, they were treated with different micromolar concentrations of Trx-rHelja and rHelja in DMEM-1% FBS. After an additional 24 h, the cultured medium was removed and 100 μL of MTT reagent (1 mg/mL in DMEM-1% FBS) were added to each well and incubated 4 h at 37 °C. Then, the solution was removed and 100 μL of dimethyl sulfoxide was used to dissolve the complex. Absorbance was measured at 570 nm using a microplate reader ELx800 (Bio-Tek Instruments, Winooski, VT, USA). The percentage of cellular viability was obtained normalizing to the values in the absence of treatment (100% viability). Each treatment was tested in triplicate and the experiment was repeated at least three times. Standard deviation (square root of variance among the three samples in each experiment), one-way ANOVA and Tukey test (*p* ≤ 0.05) analysis were performed using InfoStat software (2014, Agricultural College of the National University of Córdoba, Argentine) (available online: http://www.infostat.com.ar).

### 3.10. Microscopic Analysis

Neuroblastoma SH-SY5Y cells, treated with different concentrations of lectins, were examined by phase contrast microscopy using an Olympus CKX41 inverted microscope with a 10× lens (Richmond Hill, ON, Canada). Images were acquired with an Olympus Qcolor3 camera associated with QCapture Pro 7 software (QImaging, Surrey, BC, Canada).

## 4. Conclusions

In summary, the sunflower lectin Helja was expressed in *Escherichia coli* and its identity was verified using immunoanalysis. It was demonstrated that the protein chimera displayed toxicity on human SH-SY5Y neuroblastomas, decreased the viability of these tumor cells by 75% and simultaneously disturbed the cell morphology. The fact that reports of anti-neuroblastoma lectins are scarce confers relevance to the results, thus rHelja emerges as a promising tool for the development of diagnostic or therapeutic methods for neuroblastoma cells, the most common solid tumors in childhood.

## Supplementary Materials

Supplementary materials can be found at www.mdpi.com/1422-0067/18/1/92/s1.

## Abbreviations

CDS	coding sequence
Con A	concanavalin A
EM	extracellular matrix
Helja	*Helianthus annuus* jacalin
IPTG	isopropyl-β-d-thiogalactopyranoside
MTT	3-(4,5-dimethylthiazol-2-yl)-2,5-diphenyltetrazolium bromide
ORF	open reading frame
PBS	phosphate buffered saline
PCR	polymerase chain reaction
ROS	reactive oxygen species
