# Supplementary material for: Anti-Neuroblastoma Properties of a Recombinant Sunflower Lectin
Source: Int J Mol Sci. 2017 Jan 10;18(1):92. doi: 10.3390/ijms18010092 (PMC5297726; doi:10.3390/ijms18010092)
Supplement: Supplementary file 1 [file ijms-18-00092-s001.pdf]

Supplementary Materials: Anti-Neuroblastoma Properties of a Recombinant Sunflower Lectin

Marcela Pinedo, Melanie Genoula, María Ximena Silveyra, André De Oliveira Carvalho, Mariana Regente, Marianela Del Río, Júlia Ribeiro Soares, Valdirene Moreira Gomes and Laura De La Canal

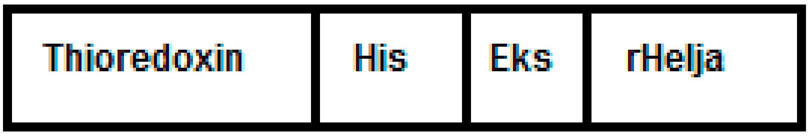

Figure S1. Recombinant protein with the fusion of Thioredoxin, hisitidine tag (His), enterokinase digestion site (Eks) and Helja.

MSDKIIHLTD DSFDTDVLKA DGAILVDFWA EWCGPCKMIA PILDEIADEY QGKLTVAKLN IDQNPGTAPK YGIRGIPTLL LFKNGEVAAT KVGALSKGQL KEFLDANLAG SGSGHMHHHHHHSSGLVPRG SGMKETAAAK FERQHMDSPD LGTDDDDK*MA NNYVEVGPWG GSGGANPWSI IPNGGRITRI NVRSGAIVDA IYFGYTEGGT NYETAIFGGR NGSLSTIDIA DDEEIIEING KVATFENLNL VTQLTFVTNK QTHGPYGTNG GTDFSCPIAK GKVVGFFGRY GAYLDAIGVV LSP*.

A-Scheme representing the recombinant protein with the fusion of Thioredoxin, hisitidine tag (His), enterokinase digestion site (Eks) and Helja.

B-Deduced amino acid sequence from the nucleotide sequencing of the pET-32 EK/LIC vector (pET System Manual, Novagen) containing Helja CDS. The sequence was provided by Unidad Genomica, a facility of the Instituto de Biotecnología-at—INTA Castelar, Argentina using the forward and reverse primers for T7. Reverse sequences were converted to the antiparallel strand, and the two readings were aligned using Clustal Omega at http://www.ebi.ac.uk/Tools/msa/clustalo/ (Sievers and Higgins, 2014). Bold letters indicate the aminoacidic sequence for thioredoxin while hisitidine tag and enterokinase digestion site are underlined and Helja sequence is indicated in italic.

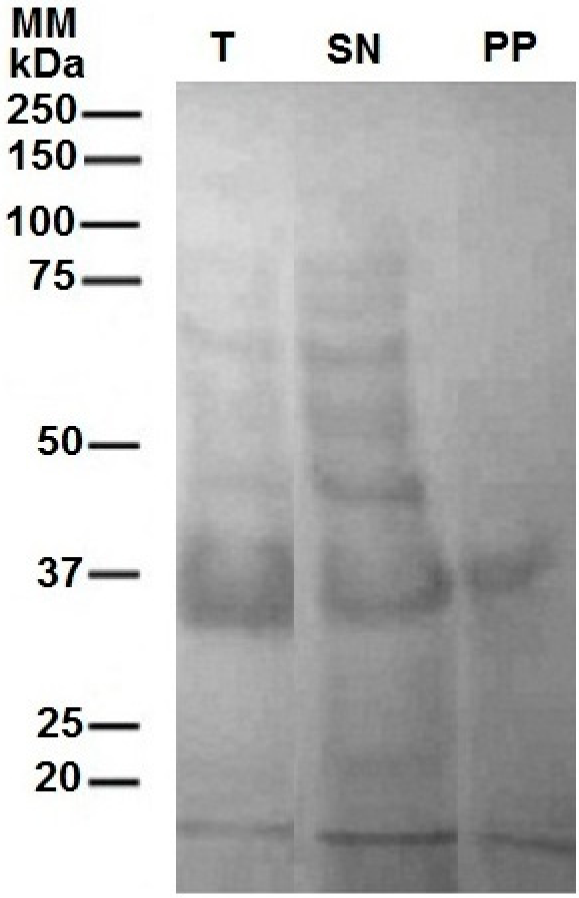

Figure S2. Protein profile of extracts obtained after lysis of IPTG induced cells cultures. The extracts (T) were centrifuged at 10.000× *g* 30 min. The supernatant (SN) and the pellet (PP) were loaded on a 12% SDS-PAGE on the base of an equivalent cell number, and subsequently stained with Coomassie Brillant Blue.
